# Connecting the dots: using a network approach to study the wellbeing spectrum

**DOI:** 10.1007/s12144-024-06363-0

**Published:** 2024-08-06

**Authors:** Anne Landvreugd, Margot P. van de Weijer, Dirk H. M. Pelt, Meike Bartels

**Affiliations:** 1 Department of Biological Psychology, Faculty of Behavioural and Movement Sciences, Vrije Universiteit Amsterdam, Amsterdam, The Netherlands; 2 Amsterdam Public Health Research Institute, Amsterdam University Medical Centre, Amsterdam, The Netherlands

**Keywords:** Wellbeing, Personality, Depression, Network, Flourishing, Happiness

## Abstract

In positive psychology, defining the wellbeing construct has been a challenge. We used the psychometric network approach to study the structure of wellbeing. The sample consisted of Dutch adults registered with the Netherlands Twin Register. The variables were measured through standardized surveys. The network was estimated using the Mixed Graphical Models method and least absolute shrinkage and selection operator (LASSO) regularization to limit the number of spurious edges. We estimated a network in a trimming sample (*N*=1343, 63% females, *M*_age_ = 53.18, *SD*_age_ = 9.45) and in an estimation sample (*N*=726, 75% females, *M*_age_ = 45.27, *SD*_age_ = 11.12) to examine its performance and accuracy. Our final network consists of a positive cluster including satisfaction with life, subjective happiness, and flourishing items, and a negative cluster including depressive symptoms, loneliness, and neuroticism items. We identified the four most central nodes: one satisfaction with life item, one neuroticism item, and two depression items. This suggests that to get a general sense of the wellbeing construct, these items would serve as most informative.  The network approach clearly demonstrates the different, yet connected positive and negative clusters of wellbeing and therefore re-affirms the complex interconnectivity of wellbeing phenotypes. In addition, the network results reject the view of strictly delineated wellbeing domains. Having identified the most central nodes in the network, these can be used in futures studies with limited resources, as they are likely to be the most representative of the wellbeing spectrum.

**Supplementary Information:**

The online version contains supplementary material available at 10.1007/s12144-024-06363-0.

## Introduction

Defining and delineating wellbeing as a construct has proven to be a difficult challenge for the field of positive psychology. Wellbeing can be considered an umbrella term for many different more or less connected constructs, which can lead to difficulties when interpreting and comparing results from positive psychological research (Linton et al., 2016).

Diener’s theory on subjective wellbeing (SWB) is grounded in the hedonistic tradition of wellbeing and proposes that SWB is comprised of life satisfaction (cognitive SWB), high levels of positive affect, and low levels of negative affect (emotional SWB) (Diener, 1984). On the other hand, Ryff’s theory on psychological wellbeing (PWB) is grounded in the eudaimonic tradition of wellbeing and states that PWB is comprised of six dimensions: autonomy, environmental mastery, personal growth, positive relationships, purpose in life, and self-acceptance (Ryff, 1989). A similar influential theory is self-determination theory (SDT) (Ryan & Deci, 2000). Central to SDT is an individual’s experience of autonomy, competence, and relatedness, which are argued to promote wellbeing. This is slightly different from Ryff’s PWB theory, where these dimensions are believed to be components of wellbeing. Combining aspects from both eudaimonic and hedonic theory, Keyes formulated a theory on flourishing that posits that wellbeing or mental health is defined by high levels of emotional, psychological, and social wellbeing, and an absence of psychopathy (Keyes, 2005). The inclusion of social wellbeing is in line with eudaimonic ideology and is well-supported by wellbeing literature (Keyes, 1998). While these theories focus on specific aspects of wellbeing, it is also possible to evaluate wellbeing in a broader context. Wellbeing is highly (phenotypically and genetically) correlated to multiple traits, such as depression, neuroticism, loneliness, and self-rated health. In previous research, these traits were collectively referred to as “the wellbeing spectrum” (WBS) (Baselmans et al., 2019).

While the previously mentioned wellbeing theories have very early origins (Ryff, 1989; Keyes, 2005), the definition and concept of wellbeing is continuously being studied in recent literature. In a paper from 2020, Intelisano, Krasko & Luhmann, aimed to create a model to describe similarities and differences among existing wellbeing and happiness theories. They proposed a two-dimensional taxonomy: the degree of stability and psychological processes. Degree of stability refers to whether wellbeing is described as a stable trait or a temporary state. The psychological process refers to whether wellbeing is described as an affective process or cognitive process. Their model combined theories from both philosophy and psychology, allowing for a more interdisciplinary definition of wellbeing (Intelisano et al., 2020). Coming from a similar point of view, Michell and Alexandrova (2021) argue that there is a strong methodological basis for well-being pluralism. Wellbeing pluralism would accommodate the richness and diversity of wellbeing, allowing us to reconcile the philosophic and scientific concept of wellbeing. In 2020, Thorsteinen and Vittersø performed factor analyses for wellbeing, and estimated a pathway model to study the convergent and discriminant validity of hedonic wellbeing and eudaimonic wellbeing. Their models concluded that wellbeing is most likely to consist of these two constructs, that are distinct but closely related at the same time (Thorsteinsen & Vittersø, 2020).

One method to study the structure of a concept such as wellbeing has emerged from network theory. Network theory advocates that symptoms are all part of an interactive system and allows us to take a closer look at item-item associations without assuming that their correlations stem from an underlying factor (Kan et al., 2020). A network consists of a set of nodes (i.e. symptoms) along with a set of specified ties (edges) linking the nodes (Borgatti & Halgin, 2011; Epskamp et al., 2018). The general aim is to characterize the structure of the network and the position of nodes, and to use the network to better understand the examined construct. The network is used to reveal which components are most “central” using the concept of centrality: components with a high degree of centrality are most strongly connected to other items (Fried et al., 2017), and are therefore thought to be most influential in the network. Following this line of reasoning, components that are central to the network (i.e., high levels of centrality), may serve as targets for the development of prevention and intervention strategies. At the same time, criticism has been expressed on the network approach, mainly focused on the stability of centrality indices and its suitability for psychological constructs (Bringmann et al., 2019, Neal & Neal, 2021). Nevertheless, the network approach provides an interesting new avenue for the investigation of the structure of wellbeing.

There are three studies using networks to investigate the underlying structure of wellbeing. Giuntoli and Vidotto estimated a network including measures of both SWB and PWB in an Italian adult sample (*N* = 2392) (Giuntoli & Vidotto, 2021). The authors conclude that the final estimated network was most in line with Diener’s wellbeing definition, with life satisfaction, positive and negative experiences, and perceived positive functioning as different but connected wellbeing domains. Van Woerkom et al. (2022) examined how fluctuations in specific components of wellbeing are associated with fluctuations in other components of wellbeing by estimating a longitudinal wellbeing (*N* = 151). Their analysis suggests that feeling satisfied is not just a component of wellbeing, but also plays an active role in triggering other related wellbeing aspects such as cheerfulness. Recently, Bjørndal et al. (2024) investigated how the items of the Satisfaction with Life Scale (Diener et al., 1985) are associated with environmental factors, such as ‘household crowding’ and ‘feeling safe when out walking’. The network showed that the items ‘my living conditions are very good’ and ‘I have gotten the most important things in my life’ were most strongly connected to the environmental factors, e.g. ‘I have trust in the public sector’ and ‘I feel that I have influence on the government’. The other two SWLS items were less connected to any of the environmental factors. All three of these network studies have provided us with new insights about connections between wellbeing and wellbeing related measures.

In line with the works by Giuntoli and Vidotto (2021) and Van Woerkom et al. (2022), we aimed to further explore the value of network theory for studying wellbeing. However, instead of only including wellbeing measures, we estimate a broad network that includes different wellbeing measures (subjective happiness, satisfaction with life, quality of life, and flourishing), as well as the broader WBS depressive symptoms (Achenbach System of Empirically Based Assessment), neuroticism (NEO Five Factor Inventory), self-rated health, and loneliness. By estimating a broad WBS network, we aim to get better insight into wellbeing in terms of how clearly delineated or interconnected wellbeing items from different domains are. Our study was conducted in a sample of Dutch adults. The network was estimated using the Mixed Graphical Models method and least absolute shrinkage and selection operator (LASSO) regularization to limit the number of spurious edges. Our study aims to investigate how network theory can enhance our understanding of the complex architecture of wellbeing. We conclude the study by considering the added value of network science as a method for answering questions about the nature of wellbeing.

## Method

### Sample

Study participants are voluntarily registered with the Netherlands Twin Register (NTR) (Ligthart et al., 2019). The NTR was established by the Department of Biological Psychology, Vrije Universiteit Amsterdam, and collects data in children (Young NTR, YNTR) and adults (Adult NTR, ANTR). For this paper we included participants from the ANTR. Every two/three years, ANTR participants are asked to fill out a survey about their personality, psychopathology, wellbeing and lifestyle. Written informed consent was obtained from all individual participants included in the study. Participants in the ANTR generally have a high social-economic status and are of Western-European decent. For the current project we used four waves: (1) the 8th wave of data collection, collected from 2008 to 2010, (2) the 10th wave, collected from 2012 to 2014, (3) the 13th wave, collected in 2017–2018, (4) and the 14th wave, collected in 2019- February 2020. These waves were selected based on availability of relevant wellbeing variables. Participants were included if they participated in at least one of these surveys. If data on multiple time-points were available, we selected the most recent time-point.

**Fig. 1:**
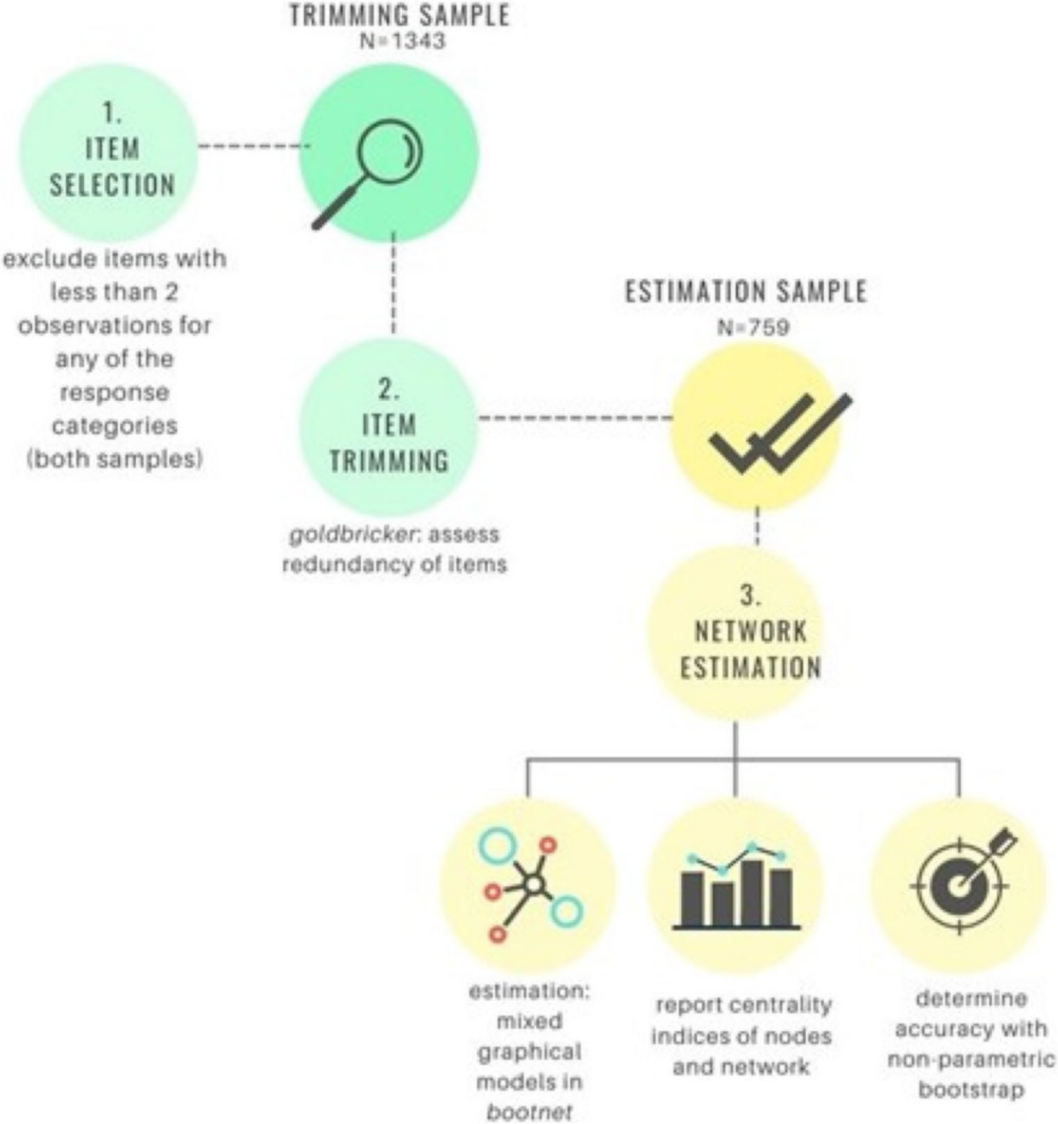
Overview of the analysis plan

Since the NTR collects data in multiples and their family members, many individuals are genetically related to each other, meaning that the observations are not entirely independent. To prevent bias due to these dependencies, we selected two samples so that within each sample, all individuals were genetically unrelated to each other. These samples were used as a trimming sample (to check for potential redundant nodes) and an estimation sample (to estimate the network) (see Fig. 1). The samples included only participants that had complete data available for all the traits. In total, the trimming sample included 1343 individuals (63% females, *M*_age_ = 53.18, *SD*_age_ = 9.45). The estimation sample included 726 participants (75% females, *M*_age_ = 45.27, *SD*_age_ = 11.12).

### Measures

The following standardized instruments were used:

- *The Subjective Happiness Scale* (Lyubomirsky & Lepper, 1999). Four items were rated on a Likert scale from 1 (strongly disagree) to 7 (strongly agree). An example of an item is: “On the whole, I am a happy person”. We recoded the items so that for all items, a higher score meant higher levels of happiness.
- *The Satisfaction with Life Scale* (Diener et al., 1985). Five items were rated on a Likert scale from 1 (strongly disagree) to 7 (strongly agree). An example of an item is: ‘My living conditions are excellent’.
- Cantril’s ladder (Cantril, 1965) was used to assess Quality of Life (QoL). Participants were asked ‘Where on the scale would you put your life in general?’, with 0 representing the worst possible life and 10 representing the best possible life.
- *The Short Flourishing Scale* (Diener et al., 2010). The scale contains 8 items that are rated from 1 to 7 using a Likert scale. 1 resembles ‘strongly disagree’ and 7 resembles ‘strongly agree’. An example of an item is: ‘I am competent and capable in the activities that are important to me’.
- *Depressive symptoms*. The depressive problems subscale from the adult self-report (ASR) of The Achenbach System of Empirically Based Assessment (ASEBA) was used to assess depressive symptoms (Rescorla & Achenbach, 2004). 14 items were rated from 0 to 2 (0 = not true, 1 = somewhat true, 2 = very true). An example of an item is: ‘I feel worthless or inferior’.
- *Loneliness*. The three items from the short scale for assessing loneliness in large epidemiological studies were used to assess loneliness (Hughes et al., 2004). For each item, participants indicated how often they identify with a statement, rated as: 0 = almost never, 1 = sometimes, or 2 = often. An example of a statement is: ‘How often do you feel isolated from others?’.
- *Neuroticism*. The NEO-FFI (NEO Five Factor Inventory) neuroticism subscale was used to assess neuroticism (Costa & McCrae, 2008). The subscale consists of 12 items, and each item was rated on a 5-point scale from 1 (strongly disagree) to 5 (strongly agree). An example of an item is ‘I often feel tense and jittery’. Half of the items were reverse-coded so that a higher score indicated higher levels of neuroticism.
- *Self-rated health*. A single item was used to evaluate self-rated health: ‘How would you rate your general health?’ (Eriksson et al., 2001). This item was rated on a 5-point scale ranging from ‘Bad’ to ‘Excellent’.

#### Statistical analyses

## Network analysis

An overview of the different steps of the analysis plan is depicted in Fig. 1.

### Item selection

Before estimating networks, we examined the item distributions. We excluded ordinal variables having less than 2 observations for any of the observed response categories as advised by (Epskamp et al., 2018).

To estimate the most parsimonious network in the estimation sample, we used the trimming sample to examine item redundancy (i.e., items that are not essential to the network since they correlate highly with other items). The goldbricker function implemented in the networktools R package (Jones, 2017) was used to assess potential item redundancy. The function operates by identifying pairs of items that are highly correlated and then flags those as redundant. With this function, strongly correlated item pairs (*r* ≥ .7) with less than 50% unique combinations with other items (i.e. less than 50% of significantly different correlations with other nodes, *p* = .05) were identified. We opted for the slightly strict 50% unique combinations criteria because we expected many items to correlate. Next, the net_reduce function was used to choose the more unique node of each redundant pair and remove the redundant one. Based on the network trimming in the trimming sample, we estimated the network without redundant nodes in the estimation sample.

### Regularized network estimation

We estimated the WBS network using the estimation sample with all items that remained after the item selection and item trimming phase. We included sex and age as covariates. The network was estimated using the *bootnet* package (Epskamp et al., 2018), and visualized using the *qgraph* package (Epskamp et al., 2012) in Rstudio (RStudio Team, 2020). Since mixed variable types (continuous and ordinal) were included in the network, the function *Mixed Graphical Models (MGM)* was chosen as the optimal regularized estimation method for our data (Haslbeck & Waldorp, 2015). Least absolute shrinkage and selection operator (LASSO) regularization with Extended Bayesian Information Criterion (EBIC) model selection was applied to limit the number of spurious edges. Due to applying LASSO, only the most significant partial correlations are retained, making the network less prone to overfitting. This method is preferred over other regularization techniques, because it can lead to parameter estimates of exactly zero (Epskamp & Fried, 2018). To avoid false positives and creating a sparse and more interpretable network, To avoid false positives, we set the EBIC tuning parameter *y* to the default value (for MGM) of 0.5. The network was plotted using multidimensional scaling (MDS), where the distance between the nodes is reflective of the strength of the association between two nodes, with nodes placed closer together sharing stronger associations.

### Centrality and clustering

We examined the centrality index *strength* (the sum of absolute edge weights connected to each node), which indicates how strongly a node is directly connected to other nodes. This measure works optimally in a network with exclusively positive edges as this index does not distinguish between positive and negative edges. We, however, expected, due to the WBS structure, positive (wellbeing, self-rated health) as well as negative (neuroticism, depression, loneliness) edges. In case of a node with both negative and positive edges, *expected influence* (EI) is a preferable measure over strength (Robinaugh et al., 2016). Therefore, we also estimated the EI of the nodes (Robinaugh et al., 2016). EI assesses a node’s influence while accounting for both negative and positive edges. Nodes with higher EI would play a bigger role in the etiology of wellbeing. For example, if the expected influence of the node “On the whole, I am a happy person” is higher than of the node “How would you rate your general health”, then the first node would be interpreted as a more important factor in overall wellbeing than the second node.

To examine the network as a whole, we estimated the *global clustering coefficient* and *local clustering coefficients* of the network. The *global clustering coefficient* (i.e. transitivity) is an estimate for how often a node’s neighbouring nodes are also connected to each other (Costantini et al., 2019). It can range between 0 and 1, with values closer to 1 indicating a highly connected and clustered network structure, while values closer to 0 indicating that the network is comprised of numerous weak ties. A highly connected network suggests a strong local interconnectedness between the variables, while a weakly connected network suggests a branched or hierarchical structure. For example, a highly connected wellbeing network would imply that all the wellbeing items strongly influence each other. Next, we calculated *local clustering coefficients* (as implemented in the *qgraph* R package) using Zhang & Horvath’s weighted clustering coefficient (Zhang & Horvath, 2005). The coefficient indicates the likelihood that a node’s neighbouring nodes (i.e. the nodes that are connected to a particular node) are also connected. A local clustering coefficient of 1 indicates that the node is at the centre of a fully interlinked cluster. This suggests the presence of tightly knit subgroups within the network. Contrarily, a coefficient of 0 indicates that a node’s neighbouring nodes are not connected at all, indicating a more sparse structure around the node.

### Edge-weight accuracy

Lastly, we examined how accurately we estimated the edge-weighs in our network. Assessing the edge-weight accuracy is important because the validity of a number of network indices (e.g. EI) is based on the edge-weights. In addition, the edges are used for the clustering indices (e.g. global clustering coefficient) and therefore the accuracy is an indicator of the reliability of these indices. This analysis was done by using the non-parametric bootstrapping in *bootnet* (Epskamp et al., 2018). Using this method, observations are resampled with replacement to create new plausible datasets where the edge-weights can be re-estimated in. Based on 1000 bootstraps, a 95% confidence interval (CI) around the edge-weights was estimated. These CIs can be used to assess accuracy of the edge-weights, with wider CIs reflecting less accurate edges.

### Factor analysis

In addition to the network analysis, we also ran exploratory (EFA) and confirmatory factor analyses (CFA), to compare the wellbeing factor structure with the estimated networks. In the EFA, we examined the number of factors to extract from the data using the “parallel” function in the *psych* package in R (Revelle, 2022). We examined how the items load on that number of factors using the ‘fa’ function, where we used minimum residual factor extraction and oblimin rotation (i.e., allowing latent factors to correlate), since we expected the different well-being components to be correlated. For the CFA, we used the network-based trimmed estimation sample to enable comparison with the network analyses. Using the *lavaan* package (Rosseel, 2012) we compare the fit of three models: (1) a six factor model with correlated factors. This model contains one factor for each included construct (excluding quality of life and self-rated health since these were removed in the trimming stage), (2) a higher-order factor model where the six factors load on one second-order “well-being spectrum” factor, and (3) a higher-order factor model where the “positive” traits (satisfaction with life, subjective happiness, and flourishing) load on a positive second-order factor, and all “negative” factors (depression, neuroticism, loneliness) load on a negative second-order factor (where the higher-order factors are allowed to correlate). Detailed methods can be found in Supplementary Materials I.

## Results

### Sample descriptives

The sample rated their mean quality of life with a 7.89 out of 10 (*SD* = 1.11). The mean general health was 3.94 out of 5 (*SD* = 0.76). The item ‘*In general*,* I consider myself a very happy person*’ was rated with a mean score of 5.99 out of 7 (*SD* = 1.00). Sample descriptives of all the separate items can be found in Supplementary Materials II.

### Item selection and trimming

Five items were removed because they did not meet the threshold of at least two observations in each category; a self-rated health item (“*How would you rate your general health?”*), three items from the flourishing scale (“*I am engaged and interested in my daily activities*”, “*I actively contribute to the happiness and wellbeing of others*”, and “*People respect me*”), and one depression item (“*I deliberately try to hurt or kill myself”).* After network trimming, four nodes were identified as redundant, i.e. strongly correlated with other nodes (*r* ≥ .7) with less than 50% unique combinations with other items. This way, 41 items were left for network estimation. More information on the redundant nodes can be found in Supplementary Materials I.

### Network structure

**Fig. 2:**
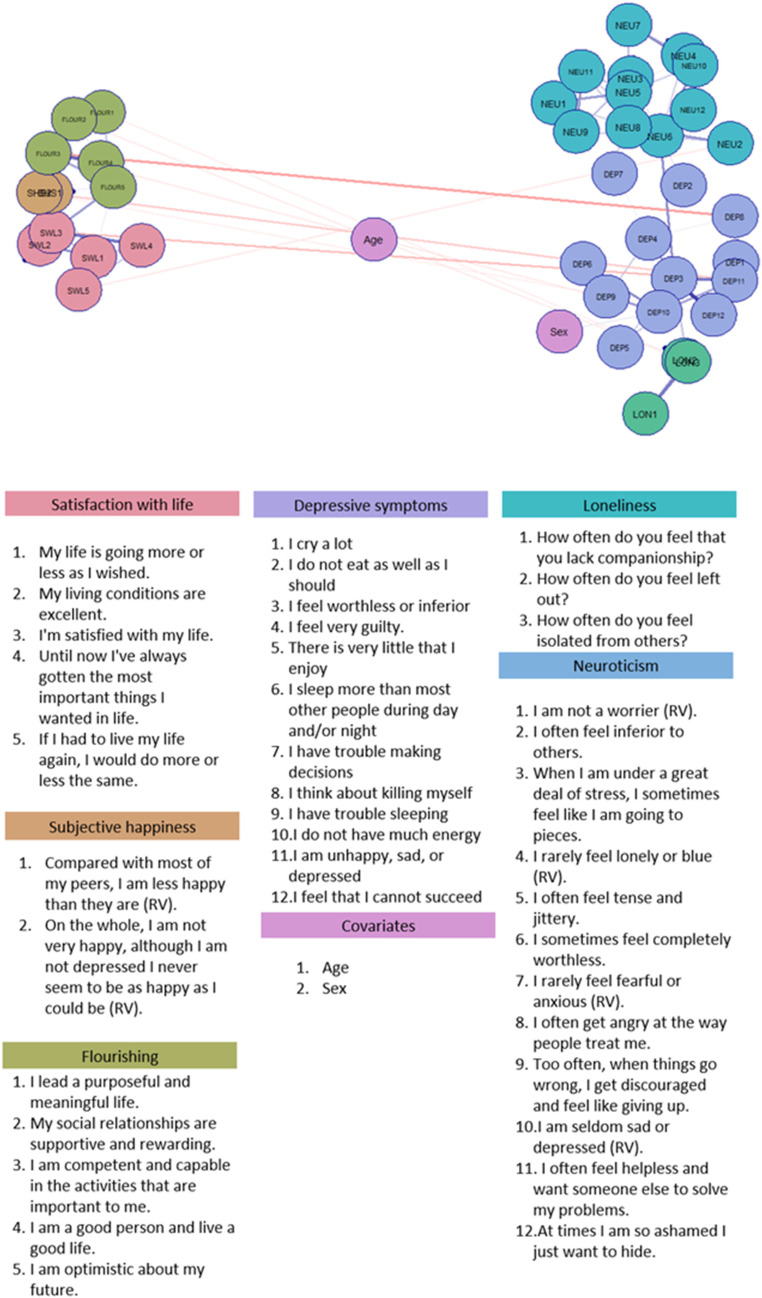
Multidimensional scaling layout network of the wellbeing spectrum. Blue lines indicate positive associations, red lines indicate negative associations. RV = reverse coded before the analyses

The multidimensional scaling (MDS) layout of the wellbeing network is shown in Fig. 2. This graph reveals two clusters: a depression, loneliness, and neuroticism cluster reflecting the more negative aspects of the WBS, and a cluster of the different wellbeing measures, reflecting the positive aspects of the WBS. Supplementary Table 1 provides the partial correlation matrix that underlies the network depicted in Fig. 2 (with item descriptions in Supplementary Table 2). The positive and negative cluster are mostly connected through depression nodes connecting to different wellbeing nodes. While loneliness and neuroticism are also directly connected to flourishing and satisfaction with life, respectively, they are mostly indirectly connected to wellbeing items through depression nodes. Additionally, we see that items of the same questionnaire tend to cluster together. Unexpectedly, Sex was barely connected to any of the variables, and only to variables in the negative cluster. Age was not connected to any of the variables.

### Centrality and clustering

**Fig. 3:**
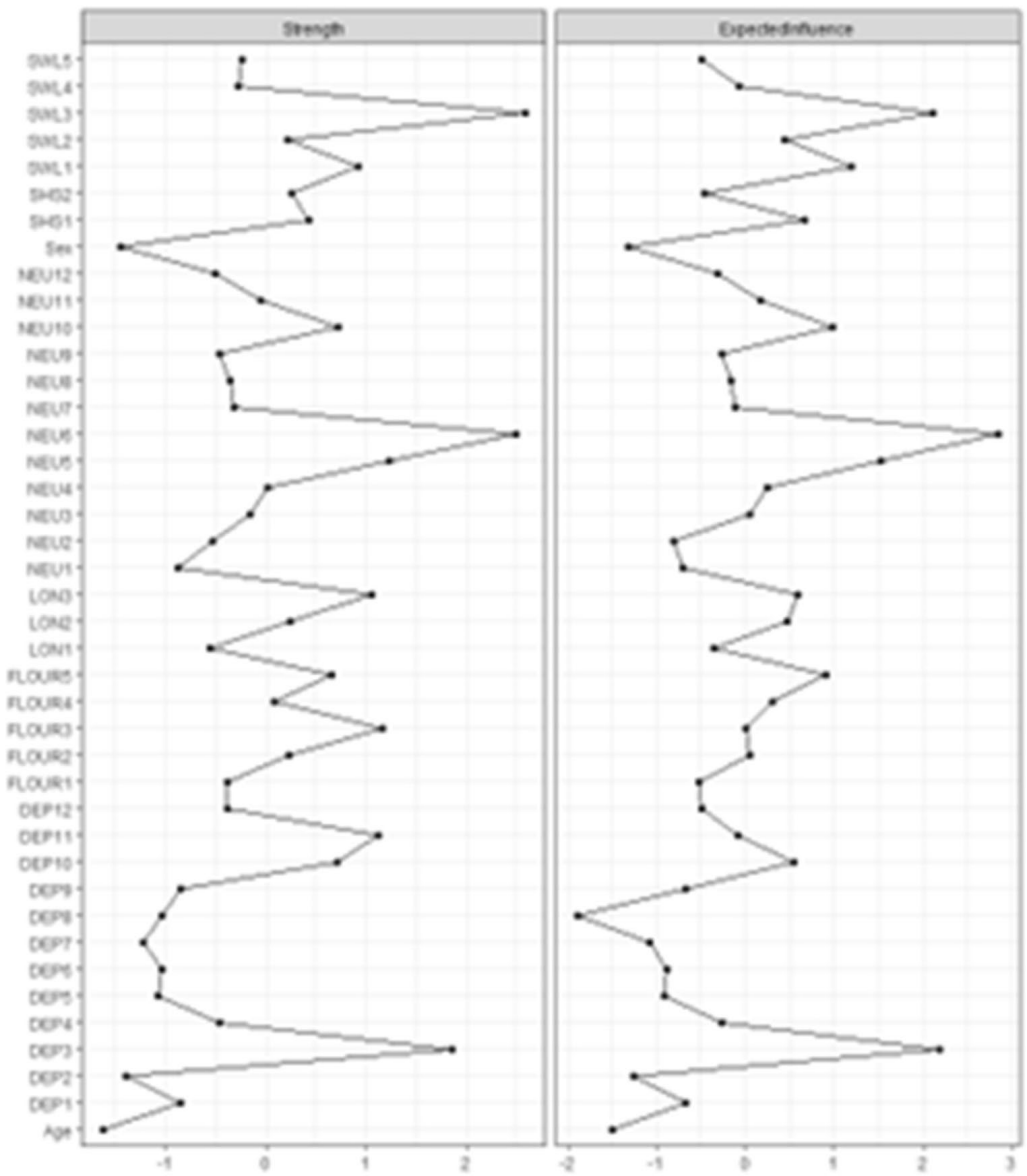
Centrality indices of all nodes (see Fig. 2 for item descriptions)

Standardized centrality indices for each item are depicted in Fig. 3. Lower, negative Z-scores indicate nodes with the least strength, while higher, positive Z-scores indicate nodes with the highest strength. The nodes that scored relatively high on strength were SWL3 (*‘I am satisfied with my life*’), NEU6 (‘*I sometimes feel completely worthless’*), DEP3 (‘*I feel worthless or inferior’*), and DEP11 (“*I am unhappy*,* sad*,* or depressed”*). This indicates that these items play the biggest role in the etiology of wellbeing. Also, this is line with the wellbeing theory of Diener (1984) because it includes both cognitive wellbeing (SWL3) and affective wellbeing (NEU6, DEP3, DEP11). Sex, which was included as a covariate, scored the lowest, meaning that sex almost plays no role in wellbeing. The results for strength and expected influence are very similar, because there were not many negative edges in the network. The global clustering coefficient of the entire network was 0.32, and the local clustering coefficients ranged from 0 to 0.38 (Supplementary Table 3).

### Edge weight accuracy

Supplementary Materials I Fig. 1 contains the bootstrapped CIs of the edge-weights (edge labels were left out for readability) that were estimated to examine the edge-weight accuracy. To achieve this, new bootstrap samples were created by sampling with replacement. For each of these bootstrap samples the edge weights were calculated. The CI’s were based on the distribution of edge weights from all the bootstrap samples. On the y-axis are all edges in the network, and on the x-axis it shows the strength of the edge-weights, with red dots as point estimates, the black dots the bootstrap means, and the grey area as 95% confidence intervals. Overall, we find relatively large CIs. These CIs do not reflect whether or not an edge should have been set at zero, but rather the accuracy of the estimated edge-weights. The results show that the strength of the edges, and therefore several network indices (e.g. EI, global clustering coefficient) should be interpreted with caution.

## Factor analysis

The EFA in our trimming sample suggested a seven-factor solution (variance explained = 47%). Within this solution (see Supplementary Table 4), items from three scales loaded exclusively on their own intended factors: neuroticism (factor 1), flourishing (factor 2), and loneliness (factor 5). Factor 3 was a composite of quality of life, self-rated health, SWL, and two SHS items. Three of the four SHS items additionally loaded on Factor 7. The 4th factor included 7 depression items, whereas the 6th factor included 3 other depression items and additionally, self-rated health loaded on this factor. In our CFA, we compared the fit of three models. We find that both the model with the single higher-order factor and the two higher-order factors fit the data significantly worse than the six-factor model without higher-order factors (see Table 1 for fit indices). Detailed results can be found in Supplementary Materials I.

## Discussion

In the present study, we set out to study the wellbeing spectrum from a network perspective to get better insight into the construct’s internal structure. The final network suggests two clusters, with on the one hand the “negative” spectrum items of depression, loneliness, and neuroticism, and on the other hand the “positive” spectrum items from the different wellbeing measures.

Visual inspection of the final network in the estimation sample suggests the presence of two smaller networks, connected through a few items. One cluster consisted of positive items for subjective happiness, satisfaction with life and flourishing, while the other, more negative, cluster included depressive symptoms, neuroticism, loneliness, and sex. The positive and negative cluster were predominantly connected by edges between multiple depression items and multiple wellbeing items, not by one or two “bridge items” (see Supplementary Tables 1–2). Age was not connected to other nodes in the network, i.e., it was independent after conditioning on all other variables, indicating that the network structure is independent of the age of the participant. Since our current sample only included adults, it would be interesting to repeat our current efforts in a sample of children/adolescents or in a sample of older adults to see if age does affect the network in such samples.

With respect to the wellbeing items, we see that items belonging to the same measurement instrument tend to cluster together, but there are also several connections between wellbeing items from different instruments. On the one hand, the clustering of wellbeing items belonging to the same measurement instrument (i.e. flourishing, satisfaction with life, and subjective happiness) is in line with theories such as Keyes’ theory on flourishing or Diener’s theory on SWB that distinguish different wellbeing domains such as cognitive wellbeing and psychosocial wellbeing (Diener, 1984; Keyes, 2005). On the other hand, it also becomes clear that all wellbeing items are highly clustered and interconnected, suggesting that the different domains are not as clearly delineated as may be claimed by different theories/factor analytic studies. Taken together, these results are very similar to previous findings of high phenotypic and genetic correlations between WBS measures, and a genomic factor model where positive and negative traits loaded on separate, but highly correlated factors (Baselmans et al., 2019). This is also similar to findings by Giuntoli and Vidotto, who conclude based on their network analyses that different SWB and flourishing components are closely related constructs (Giuntoli & Vidotto, 2021). It is also in line with other studies emphasizing that different wellbeing phenotypes are highly interconnected (Bartels & Boomsma, 2009; Kim et al., 2016; Kokko et al., 2013).

One of the advantages of network psychometrics is the possibility to examine nodes in terms of their individual strength in the network. Four items scored relatively high compared to all other nodes: SWL3 (*‘I am satisfied with my life*’), NEU6 (‘*I sometimes feel completely worthless’*), DEP3 (‘*I feel worthless or inferior’*), and DEP11 (“*I am unhappy*,* sad*,* or depressed”*). This indicates that these nodes have stronger connections to other nodes in the network. Similarly to SWL3, Van Woerkom et al. (2022) and Giuntoli and Vidotto (2021) identified ‘feeling satisfied’ as a item that strongly influences other wellbeing items. NEU6, DEP3, and DEP11 had not been identified as highly influential components of the wellbeing network yet, and therefore provide us with new insight in the structure of wellbeing. A potential interpretation is that the most central nodes reflect the items that are most representative of the WBS. Examining these items in the factor analytic context, these are also items with high communalities compared to the rest of the items (see Supplementary Table 5). Thus, to get a general idea of an individual’s wellbeing with limited resources, one might benefit most from examining these items.

As any approach, the network approach has its limitations. A common critique is the validity of the centrality indices in the context of psychological networks. Bringmann and colleagues (2019) provide three reasons for why centrality indices might not be suitable for psychological networks. First, the indices were originally developed for social networks (where connections are direct representations of raw data) but these are substantially different from psychological networks (where connections are coefficients derived from a model). Second, some indices (especially closeness and betweenness) have shown to be unstable in psychological networks. This instability might occur because the centrality indices are susceptible to which nodes are included in the network, and it is unknown which nodes should be included beforehand (Neal & Neal, 2021). Third, there has been little research on the predictive power of centrality indices in psychological networks. Whether centrality indices can thus point to clinically relevant symptoms as targets for intervention is not entirely clear.

Besides being subjected to these general critiques of network analysis, our findings should be interpreted in the context of a number of other limitations. First, we found that items that belong to the same questionnaire tend to cluster together. While this partly reflects these items successfully capturing a particular construct, this likely also reflects participants answering questions belonging to the same measurement instrument more similarly than questions from different instruments as these items were presented with the same response format (Weinberger et al., 2006). The response format (e.g., scale, wording) differ across questionnaires, potentially leading to clustering. Second, items in a questionnaire are often designed to load on one certain scale and not on another scale, e.g. through factor analysis. This way of designing items could be interfering with the actual underlying correlations between the items of different scales. Third, we were limited by the wellbeing items that were previously collected in our sample. For example, we did not include Ryff’s different scales or items corresponding to Keyes’ social wellbeing domain. This relates to the boundary specification problem, referring to the difficulty of deciding which nodes should be included when estimating a network (Neal & Neal, 2021). Ideally, all possible nodes should be included in a network, but it is impossible to know the boundary of the theoretical network, and so we are bounded by what we have measured. Importantly, this issue is not unique to network analyses, but also applies to factor analytic studies. Nevertheless, in future research even broader wellbeing networks including items based on these different theories need to be estimated. In addition, future research should compare wellbeing networks in different ethnic and age groups, to see how the network dynamics is different in different populations.

## Conclusions

The results described in this study support previous research on the WBS that links different items assessing wellbeing, depression, neuroticism, and loneliness to form two highly interconnected positive and negative clusters (Baselmans et al., 2019). Additionally, we identify four nodes most central to the network: one life satisfaction item, one neuroticism item, and two depression items. This suggests that to get a general sense of the WBS, these items would serve as the most informative items to evaluate. Wellbeing research with limited resources should therefore focus on these four items to get the most accurate and comprehensive understanding of wellbeing. Nevertheless, taking a network perspective re-affirmed prior research that demonstrates the complex interconnectivity of different wellbeing (related) phenotypes. While several items definitely cluster with other items within the same construct, the network results also reject the view of clearly delineated wellbeing domains. To develop a more complete picture of wellbeing, including hedonic and eudaimonic aspects in a network context, additional studies are needed that include more wellbeing measures that measure these different aspects of wellbeing. Nevertheless, taking a network perspective re-affirmed prior research that demonstrates the complex interconnectivity of different wellbeing (related) phenotypes. While several items definitely cluster with other items within the same construct, the network results also reject the view of clearly delineated wellbeing domains. To develop a more complete picture of wellbeing, including hedonic and eudaimonic aspects in a network context, additional studies are needed that include more wellbeing measures that measure these different aspects of wellbeing.

## Electronic supplementary material

Below is the link to the electronic supplementary material.

Supplementary Material 1

Supplementary Material 2

Supplementary Material 3

Supplementary Material 4

## Data Availability

The Netherlands Twin Register cohort data may be accessed through the Netherlands Twin Register repository (ntr.fgb@vu.nl) upon approval of the data access committee.
