# Supplementary figures and images for: Connecting the dots: using a network approach to study the wellbeing spectrum
Source: Curr Psychol. 2024 Aug 6;43(34):27365–76. doi: 10.1007/s12144-024-06363-0 (PMC11420360; doi:10.1007/s12144-024-06363-0)

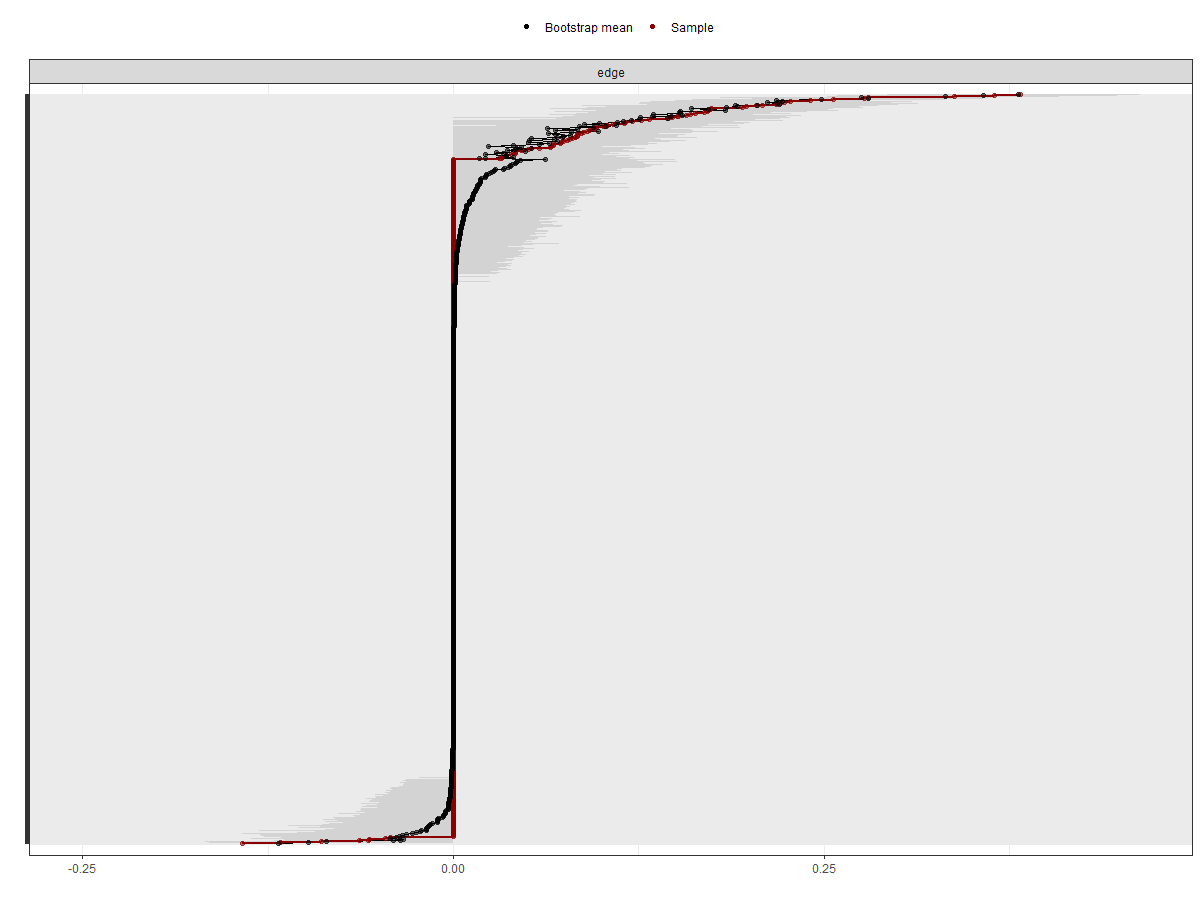

Supplement: Supplementary file 1 — Supplementary Material 1 [file 12144_2024_6363_MOESM1_ESM.png]
