# Supplementary material for: Connecting the dots: using a network approach to study the wellbeing spectrum
Source: Curr Psychol. 2024 Aug 6;43(34):27365–76. doi: 10.1007/s12144-024-06363-0 (PMC11420360; doi:10.1007/s12144-024-06363-0)
Supplement: Supplementary file 3 — Supplementary Material 3 [file 12144_2024_6363_MOESM3_ESM.docx]

**Connecting the dots: Using a network approach to study the wellbeing spectrum**

**Supplementary material**

**Network trimming**

Four nodes were identified that could be removed in the redundancy check. The quality of life item (*“Where on the scale would you put your life in general?”*) was deemed redundant since it was not significantly different from a satisfaction with life item (“*I'm satisfied with my life*”*)* within the context of this network. Two subjective happiness scale items (*“On the whole I am a happy person”* & *“On the whole, I am very happy, I enjoy life come what may and I always make the best of things”*) were excluded because of redundancy in the context of two other subjective happiness items: “*Compared with most of my peers, I am less happy than they are”* and “*On the whole, I am not very happy, although I am not depressed I never seem to be as happy as I could be*”, respectively. Lastly, one depression item (*“I feel tired without good reason”*) had a redundant role in the network because of another depression item (“*I do not have much energy*”). After removing these items from the estimation data, 41 items (including covariates) were left for network estimation.  Importantly, the exclusion of these items does not mean they are unrelated to the network, but rather that the item is redundant for this specific network because there is another item that plays a similar role in the network. For example, one of the satisfaction with life items was highly correlated with the excluded quality of life item (*r* = .73) and additionally correlated similarly with other nodes in the network. Since this satisfaction with life item was more unique to the network than the quality of life item (in terms of its redundancy statistics with other nodes), this item was retained instead of the quality of life item. This does not mean that quality of life should be disregarded with respect to wellbeing, but rather that it correlates similarly with the rest of the network as satisfaction with life

**Factor analysis**

**Introduction**

The most common way in which this the well-being construct has been studied is through factor analytical methods. In these models, item responses are modelled so that they “load” onto higher-order well-being factors such as subjective well-being and psychological well-being, and the relation between these higher-order factors is evaluated by correlating them with each other. (Robinaugh et al., 2020). The main conceptual difference with network analysis is that factor analysis is founded on the idea of a common latent factor (e.g., well-being) that causes the related “symptoms” (e.g., life satisfaction aspects). Contrarily, network theory advocates that symptoms are all part of an interactive system. So far, factor studies have provide mixed results in terms of the structure of well-being, with some studies finding single factor solutions (Kim et al., 2016), and some finding multiple-factor solutions with varying degrees of correlations between these factors (Joshanloo, 2016; McMahan & Estes, 2011; Vanhoutte & Nazroo, 2014). Factor analytical methods implicitly assume a top-down (reflective) model in which correlations between indicators are explained by the latent factor. This means that they assume that conditional on the latent factor, residual correlations between the items are zero. Consequently, information on the associations between the different items, independent from them loading on the same higher-order factors, is lost. Therefore, by modeling well-being items as part of an overarching construct, we risk losing important information on the relation between these different components at the item level.

**Methods**

For comparison purposes, we used the trimming and estimation samples to run exploratory (EFA) and confirmatory factor analysis (CFA), respectively. First, in EFA, we examined the number of factors to extract from the data using the “parallel” function in the *psych* package in R (Revelle & Revellle, 2022). We examine how the items load on that number of factors using the ‘fa’ function, where we use minimum residual factor extraction and oblimin rotation, since we expect the different well-being components to be correlated. We examine potentially redundant items based on their communalities (i.e. the proportion of an item’s variance that can be explained by the factors). As a threshold, a communality of over .3 was deemed acceptable. We use this threshold to compare which items are left out of the network analysis to those left out based on factor analysis.

To enable comparison with the network analyses, we use the network-based trimmed estimation sample for our CFA. In this reduced set of items we assume that all items load on a factor representing their corresponding construct. Using the *lavaan* package (Rosseel, 2012) we compare the fit of three models: 1) a six factor model with correlated factors. This model contains one factor for each included construct (excluding quality of life and self-rated health since these were removed in the trimming stage) , 2) a higher-order factor model where the six factors load on one second-order “well-being spectrum” factor, and 3) a higher-order factor model where the “positive” traits (satisfaction with life, subjective happiness, and flourishing) load on a positive second-order factor, and all “negative” factors (depression, neuroticism, loneliness) load on a negative second-order factor (where the higher-order factors are allowed to correlate). We compare the fit of the higher-order models to the fit of the six-factor model without higher-order factors using a likelihood ratio test for comparing nested lavaan models.

*Comparison of the network model and the factor model* After fitting both models, we compared the results in two ways. First, we examined which items were excluded from the well-being network based on the redundancy, and compared this to the items that were excluded based on communalities in the EFA. Second, we compared the structure of the WBS based on the network approach and the factor analytic approach.

**Results**

We exploratively examined the factor structure of our items using EFA in our trimming sample. Parallel analysis suggested an nine-factor solution. A closer examination of the extracted factor solution indicated that only two items had factor loadings >.3 on the last two factors, and that both these factors explained only 1% of the variance. Therefore, a seven-factor solution seemed a more sensible solution (variance explained = 47%). Within this solution (see Supplementary Table 4), items from three scales loaded exclusively on their own intended factors: neuroticism (factor 1), flourishing (factor 2), and loneliness (factor 5). Factor 3 was a composite of quality of life, self-rated health, SWL, and two SHS items. Three of the four SHS items additionally loaded on Factor 7. The 4^th^ factor included 7 depression items, whereas the 6^th^ factor included 3 other depression items and additionally, self-rated health loaded on this factor. Moreover, in the seven-factor solution, two neuroticism items and six depression items had a communality lower than .3 (see Supplementary Table 5).

In our confirmatory factor analysis, we compared the fit of three models: a model with six correlated factors corresponding to the six well-being constructs, a model where we include one higher order well-being spectrum factor on which the six latent factors load, and a model where we include one higher order factor for positive traits, and one for the negative traits. We find that both the model with the single higher-order factor and the two higher-order factors fit the data significantly worse than the six-factor model without higher-order factors (see Table 1 for fit indices). In the six-factor model, all correlations between the latent factors were of medium to high strength, with absolute correlations ranging between .52 and .86 . An overview of the correlations between all factors can be found in Supplementary Table 6.

| **Table 1** |  |  |  |  |  |  |  |  |  |
| --- | --- | --- | --- | --- | --- | --- | --- | --- | --- |
| Model fit comparisons | | | | | |  |  |  |  |
|  | ***df*** | **AIC** | **BIC** | **CFI** | **RMSEA** | ***χ*^2^** | **Δ*χ*^2^** | **Δ *df*** | ***p*-value** |
| Six factor model | 687 | 58061 | 58490 | .915 | .046 | 1789.5 |  |  |  |
| One higher-order factor | 696 | 58171 | 58559 | .906 | .049 | 1917.9 | 128.46 | 9 | <2.2x10^-16^ |
| Two correlated higher-order factors | 695 | 58462 | 58462 | .914 | .047 | 1814.9 | 25.459 | 8 | .001 |
| *Note.* We compare the six-factor model to the two other models. | | | | | | | | | |

*Comparison of the network model and the factor model*

There are two comparisons we can make between our network and factor model. First, we used the trimming sample to exclude possibly redundant items based on item-item correlations in the network analysis, and to examine which items were badly captured by the factors in the EFA. Thus, for the former, we exclude items based on the fact that they are almost fully captured by other items (i.e., redundant) in the network. In contrast, the latter excludes items that do not seem to fit in with the rest of the items based on the common variance between items. Unsurprisingly, these two opposing strategies lead to very different outcomes: whereas the network trimming leads to the exclusion of mostly well-being items, the factor strategy leads to the exclusion of neuroticism and depression items. Since redundancy in network analysis is based on high correlations between different items, we find that items deemed redundant in the network analysis have relatively high communalities in the factor analysis (between .469 and .750).

Second, we used the trimmed sample (with item exclusion based on the network trimming) to examine the structure of the WBS based on network estimation and CFA. The network analysis indicates that the different items are clustered within their own construct but were simultaneously highly interconnected across different constructs. Moreover, we found that the more items belonging to more positive phenotypes clustered on one side of the network, while the items belonging to more negative phenotypes clustered on the other side of the network. For the factor analysis, on the other hand, we found that six separate factors corresponding to the separate constructs were a better fit to the data than a factor model with one or two higher-order factors.

**Conclusion**

We also ran exploratory and confirmatory factor analyses to examine the structure of the well-being spectrum. The exploratory factor analyses showed that some factors clearly represented a distinct well-being construct, while others were more mixed. This is in line with our network results where most items clustered within their own construct but were also interconnected across different constructs. Moreover, our confirmatory analyses indicated that six independent but moderately to strongly correlated factors were a better fit for the data than a model with one or two overarching well-being factors. In contrast to the confirmatory factor analysis, our network approach suggests that the well-being items form a connected system consisting of a positive and negative side. While it is not possible to determine whether the factor or network structure is a better depiction of reality based on these results, it is interesting to see how both approaches provide us with different but complementary information.

**References**

Joshanloo, M., 2016. Revisiting the Empirical Distinction Between Hedonic and Eudaimonic Aspects of Well-Being Using Exploratory Structural Equation Modeling. Journal of Happiness Studies 17, 2023–2036. https://doi.org/10.1007/s10902-015-9683-z

Kim, K., Lehning, A.J., Sacco, P., 2016. Assessing the factor structure of well-being in older adults: findings from the National Health and Aging Trends Study. Aging & mental health 20, 814–822. https://doi.org/10.1080/13607863.2015.1037245

McMahan, E.A., Estes, D., 2011. Hedonic Versus Eudaimonic Conceptions of Well-being: Evidence of Differential Associations With Self-reported Well-being. Social Indicators Research 103, 93–108. https://doi.org/10.1007/s11205-010-9698-0

Revelle, W., Revellle, M.W., 2022. Package “psych.” The comprehensive R archive network.

Robinaugh, D.J., Hoekstra, R.H.A., Toner, E.R., Borsboom, D., 2020. The network approach to psychopathology: a review of the literature 2008–2018 and an agenda for future research. Psychological Medicine 50, 353–366. https://doi.org/10.1017/S0033291719003404

Rosseel, Y., 2012. lavaan: An R Package for Structural Equation Modeling. Journal of Statistical Software 48, 1–36. https://doi.org/10.18637/jss.v048.i02

Vanhoutte, B., Nazroo, J., 2014. Cognitive, affective and eudemonic well-being in later life: Measurement equivalence over gender and life stage. Sociological Research Online 19. https://doi.org/10.5153/sro.3241
